# Influence of Co concentration on properties of NiO film by sparking under uniform magnetic field

**DOI:** 10.1038/s41598-020-72883-x

**Published:** 2020-09-24

**Authors:** Posak Tippo, Wiradej Thongsuwan, Orawan Wiranwetchayan, Tewasin Kumpika, Ekkapong Kantarak, Pisith Singjai

**Affiliations:** 1 Department of Physics and Materials Science, Faculty of Science, Chiang Mai University, Chiang Mai, 50200 Thailand; 2 Graduate School, Chiang Mai University, Chiang Mai, 50200 Thailand; 3 Center of Excellence in Materials Science and Technology, Chiang Mai University, Chiang Mai, 50200 Thailand

**Keywords:** Materials science, Optics and photonics, Physics

## Abstract

Nickel oxide (NiO) films cover numerous electronic applications, including transparent conducting oxides and hole transport layer, because of its high transparency and wide band gap. A sparking discharge is a new and unique method for the deposition of NiO films due to non-complex operation and non-requirement of a vacuum atmosphere. Unfortunately, NiO films by the sparking method display a porous surface with inferior crystallinity. By assisting a uniform magnetic field in the sparking method, the porous and the crystallinity of NiO are improved. However, electrical properties of the NiO films deposited by this strategy are poor. In order to improve the electrical properties of NiO, a substitutional of Ni ions by Co ions is considered. In this study, we report an influence of Co concentration on properties of NiO films by sparking under a uniform magnetic field. Our results indicate that an increase in Co concentration to 0.1 M improves the crystallinity and increases a carrier concentration of NiO, resulting in a reduction of the resistivity. This consequence is in agreement with the increase in a number of higher-valence Ni^3+^ because of the Co^2+^ substituted Ni^2+^. Based on our research, Co-NiO film is promising materials for a transparent conductor.

## Introduction

Nickel oxide (NiO) is essentially transition metal oxides, which has been intensively studied due to its high transparency and wide band gap^1,2^. Therefore, the NiO is applied in various applications such as a gas sensor^2^, hole transport layer^3^, capacitor^4^, electrochromic^5^ and photodetector^6^. Additionally, the NiO has also found widespread applications in a heterojunction as well^7,8^. Nevertheless, using the NiO as a transparent conducting oxide (TCO) is still rare and challenging due to an inverse relationship between electrical conductivity and transparency^9,10^. Presently, the NiO films are deposited by chemical vapor deposition (CVD), sputtering and electron beam evaporation, which are required not only a vacuum atmosphere but also an oxygen source^11–13^. Thus, an alternative method for the deposition of NiO film is desired. A sparking method, consisting of a non-complex apparatus, is able to operate without a vacuum atmosphere and well deposit with various substrates^14–16^.

Recently, the deposition of NiO films by the sparking method has reported by our research group^17,18^. Their reports illustrate that the morphology of NiO films consists a lot of pores due to the agglomeration of NiO nanoparticles. To reduce the porousness of NiO films, the spark discharge was assisted by a uniform magnetic field, which reported by our previous work^19^. The effect of the uniform magnetic field on the spark discharge not only reduced the porous but also improved crystallinity of NiO films. Moreover, optical band gaps of NiO films are able to control by varying a magnitude of the uniform magnetic field. However, electrical properties of the NiO films by sparking under the uniform magnetic field are poor. In order to improve the electrical properties of the NiO films, a substitution of Ni ions by Co ions is introduced^20,21^. Our assumption believes that a lower number of the electrons in 3d level of Co^2+^ help to enhance a hole concentration of NiO films, resulting in a decrease of the resistivity.

Our work has focused on the influence of Co concentration on the properties of NiO films. The NiO films were deposited by sparking under the uniform magnetic field and its properties were modified by spin coating of Co solution at the different concentrations. The morphology, crystallinity and optical properties of Co-NiO films were characterized by an electron microscope, X-ray diffraction and UV–Vis spectrophotometer, respectively. Furthermore, the elemental compositions of films were also confirmed with an X-ray photoelectron spectrometer. To investigate the electrical properties of Co-NiO films, Hall effect along with a van der Pauw method was used.

## Results and discussion

### Morphology and crystallinity of Co-NiO films

**Figure 1:**
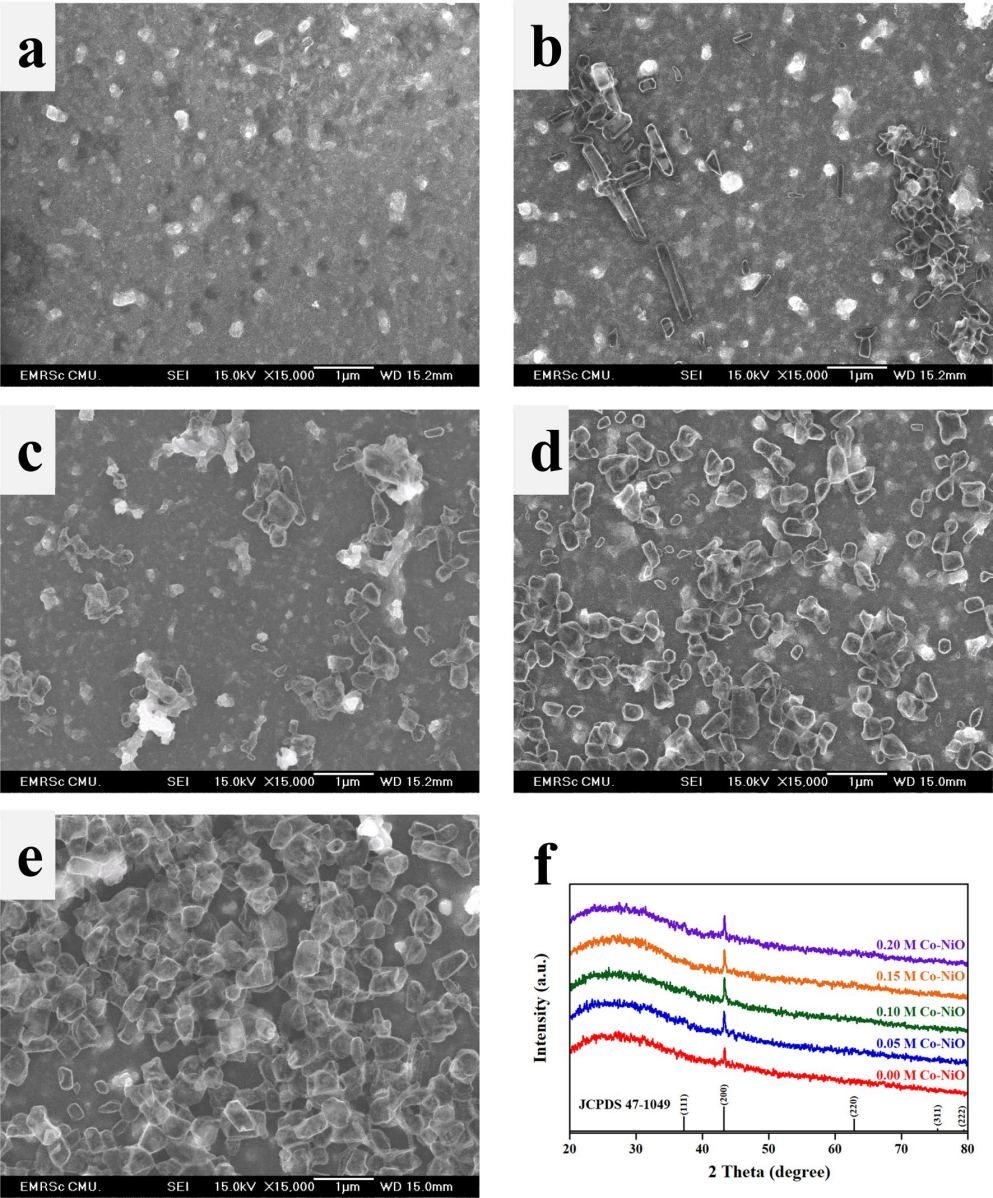
(**a**–**e**) SEM images of 0 M, 0.05 M, 0.1 M, 0.15 M and 0.2 M Co-NiO films. (**f**) XRD spectra of samples on glass substrate.

**Figure 2:**
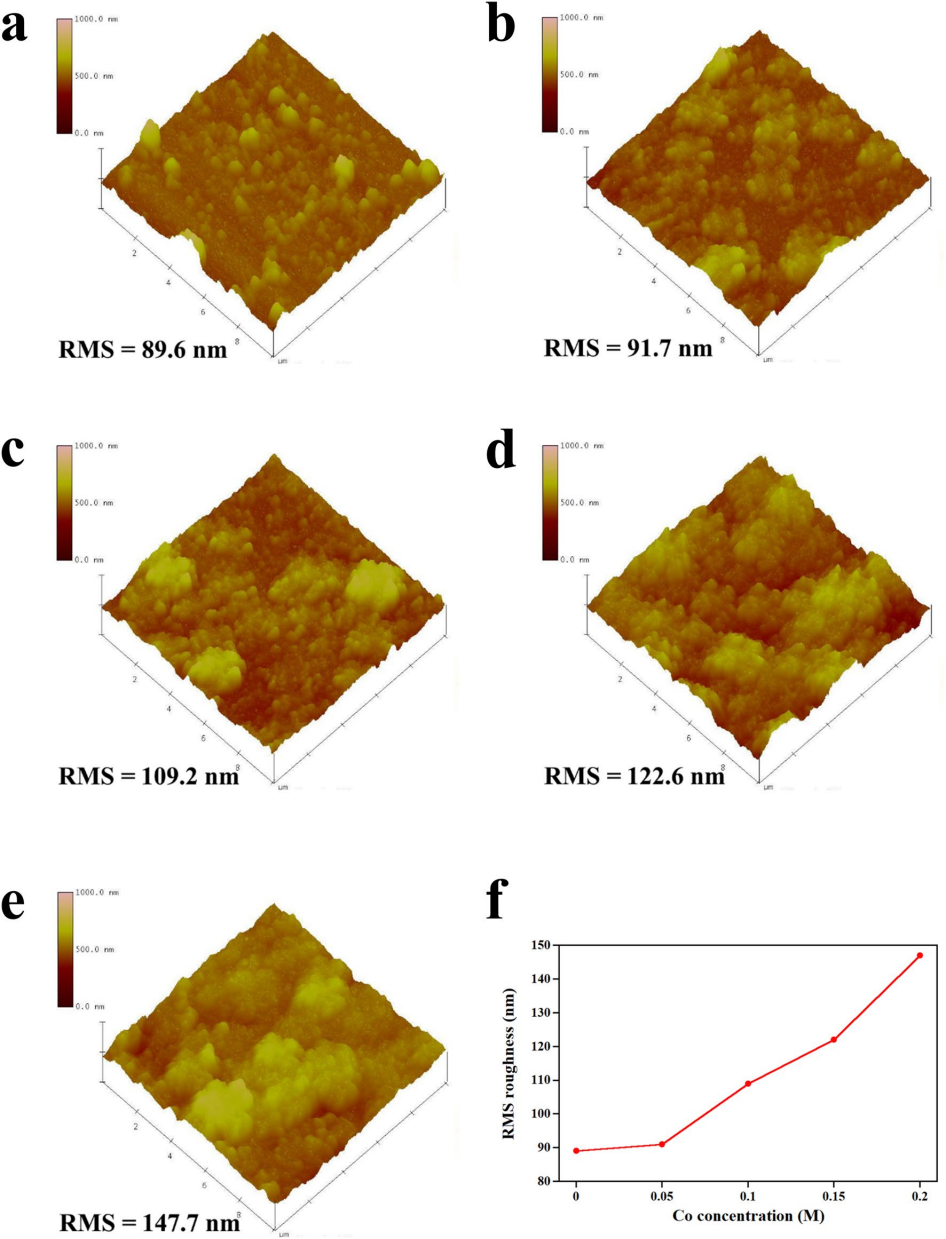
AFM images and corresponding root-mean-square (RMS) values of (**a**) 0 M, (**b**) 0.05 M, (**c**) 0.1 M, (**d**) 0.15 M and (**e**) 0.2 M Co-NiO film. (**f**) RMS roughness versus Co concentration.

Figure 1a–e indicate the SEM images of Co-NiO films at various concentrations. The formations of an irregular shape and a non-uniform size of submicron particles at the surface of NiO films are observed in Fig. 1b–e. Especially in Fig. 1b, submicro-rod structures are seen. However, the higher concentration of Co demonstrates that the submicron particles become smaller and more uniform. In Fig. 1f according to JCPDS No. 47-1049, XRD patterns of samples display a sharp peak at 43.32°, which is attributed to the (200) plane with the d-spacing of 0.209 nm and indexed to cubic symmetry with a space group of Fm-3m. By increasing the concentration of Co higher than 0.1 M, the intensity of peaks is significantly decreased. This result can be interpreted that the bigger Co ions substitute Ni ions in the NiO lattice, causing the formation of defects in the crystal structure. In order to further study the crystallinity and defect of films, we use the following equation^19^:

1 $$ D = 0.9\lambda /\beta \cos \theta $$

2 $$ \delta = \frac{1}{{D^{2} }} $$

where *D* is the crystallite size, $\lambda$ is the wavelength of X-ray (1.5418 Å), $\beta$ is the full width at half maximum (FWHM) of the diffraction peak, $\theta$ is Bragg angle of the diffraction peak and $\delta$ is the dislocation density. By deriving from (1), the *D* of 0 M, 0.05 M, 0.1 M, 0.15 M and 0.2 M Co-NiO films are calculated to be 39.6 nm, 40.2 nm, 48.3 nm, 29.6 nm and 30.6 nm, respectively. Moreover, the $\delta$ of 0 M, 0.05 M, 0.1 M, 0.15 M and 0.2 M Co-NiO films are 6.37 × 10^14^ lines m^−2^, 6.19 × 10^14^ lines m^−2^, 4.28 × 10^14^ lines m^−2^, 1.14 × 10^15^ lines m^−2^ and 1.07 × 10^15^ lines m^−2^, respectively. By the incorporation of the Co until 0.1 M, the crystallite size increased while the dislocation density decreased. In contrast, too much concentration of the Co (> 0.1 M) lowered the crystallite size and increased the defect. The reason for this will be explained in the XPS result. For the application to TCO, the RMS roughness of Co-NiO films is an essential factor. To investigate the RMS roughness of samples, the measurement via an AFM was operated. As displayed in Fig. 2, the RMS roughness of Co-NiO films increases along with the increase in the concentration of Co due to the agglomeration of excess submicron particles.

### Optical properties of Co-NiO films

**Figure 3:**
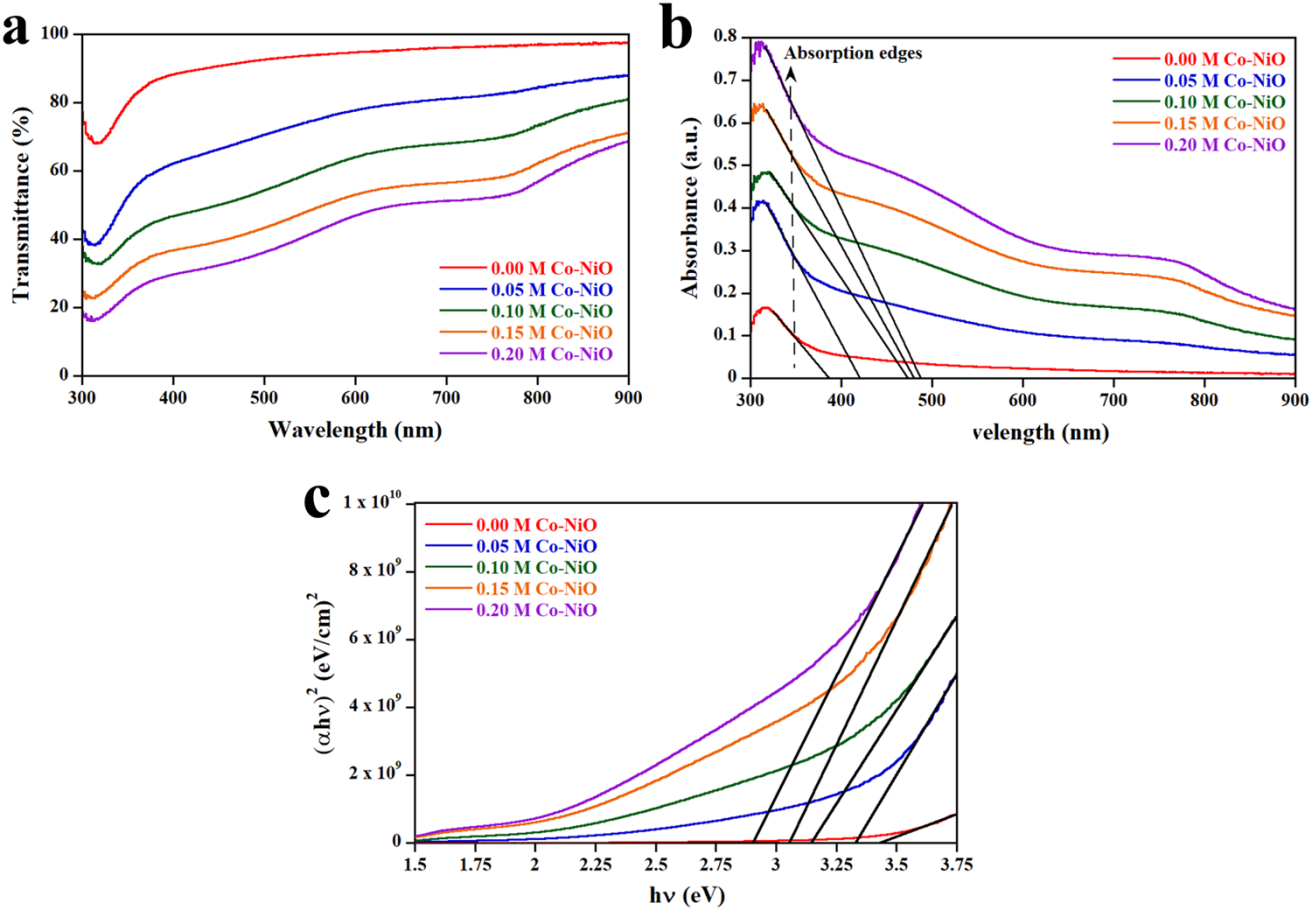
UV–Vis spectra of 0 M, 0.05 M, 0.1 M, 0.15 M and 0.2 M Co-NiO films on glass substrate. (**a**) Transmission spectra of samples. (**b**) Absorption spectra of samples. (**c**) Tauc plot of samples.

The transmission spectra of samples are displayed in Fig. 3a. The average transmittances (T_avg_) of 0 M, 0.05 M, 0.1 M, 0.15 M and 0.2 M Co-NiO films are 93.32%, 73.54%, 58.73%, 47.81% and 41.34%, respectively. By using the ellipsometer, the thickness of 0 M, 0.05 M, 0.1 M, 0.15 M and 0.2 M Co-NiO films were measured to be 419 nm, 437 nm, 481 nm, 492 nm and 534 nm, respectively. As a result, the reduction of the T_avg_ is related to the increase in the thickness of films and the reflection of an incident light caused by submicron particles. Based on this result, films prepared by our method have a better T_avg_ than films prepared by a spray pyrolysis method due to an effect of crystallinity on transmittance^21^. The sparking under the uniform magnetic field can produce films with better crystallinity for the (200) plane, resulting in a higher T_avg_ compared with the spray pyrolysis method. However, the increase in the concentration of Co indicates that the T_avg_ of our films is lower than the films (T_avg_ = 85%) prepared by the spray pyrolysis method because of the effect of the submicron particles as mentioned above.

The absorption spectra are shown in Fig. 3b. It has been observed that the increase in Co concentration not only increases the absorption intensity but also indicates the redshift of the absorption edges. This shift can be interpreted that the Ni^2+^ in the NiO lattice is substituted by Co^2+^, which reduces the band gap of the NiO film and increases the concentration of holes due to the Co^2+^ having a lower number of electrons in 3d level (3d^7^) than Ni^2+^ (3d^8^). To further verify the optical band gaps of the samples, we use Tauc’s relation expressed as^19^:

3 $$ \left( {\upalpha hv} \right) = A\left( {hv - E_{g} } \right)^{n} $$

where *h* is Planck's constant, *ν* is the photon's frequency, *A* is a proportionality constant, *E*_*g*_ is the band gap, *α* is the absorption coefficient and *n* is equal to 2 or 1/2 for indirect and direct transitions. By deriving from Tauc’s relation as shown in Fig. 3c, the optical band gaps of 0 M, 0.05 M, 0.1 M, 0.15 M and 0.2 M Co-NiO films were evaluated to be 3.45 eV, 3.33 eV, 3.15 eV, 3.06 eV and 2.91 eV, respectively. This result is consistent with the redshift of the absorption spectra.

### Chemical state of Co-NiO film

**Figure 4:**
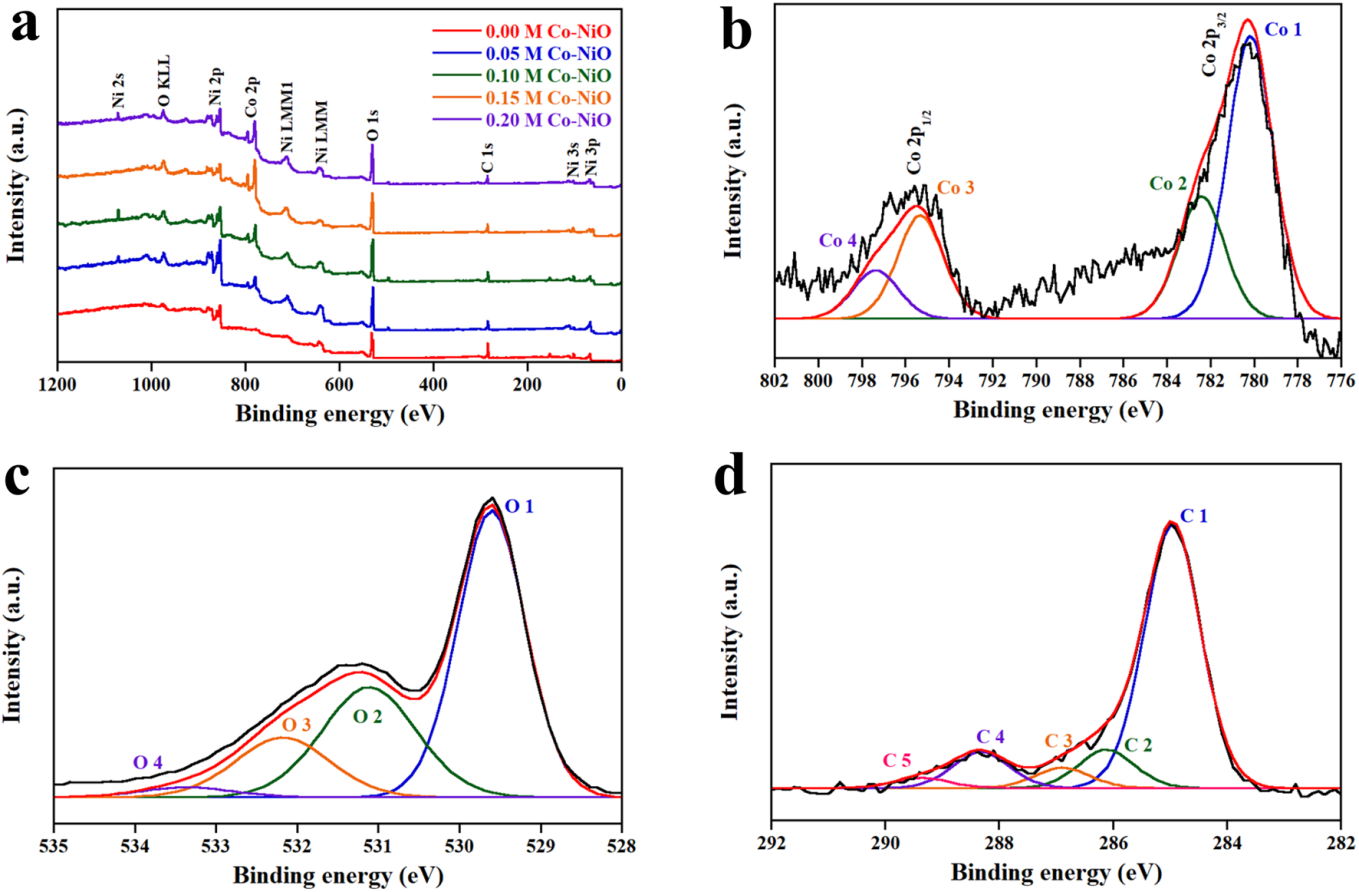
XPS spectra of 0 M, 0.05 M, 0.1 M, 0.15 M and 0.2 M Co-NiO films. (**a**) Survey scan of samples. (**b**) Co 2p core level spectrum. (**c**) O 1s core level spectrum. (**d**) C 1s core level spectrum.

**Figure 5:**
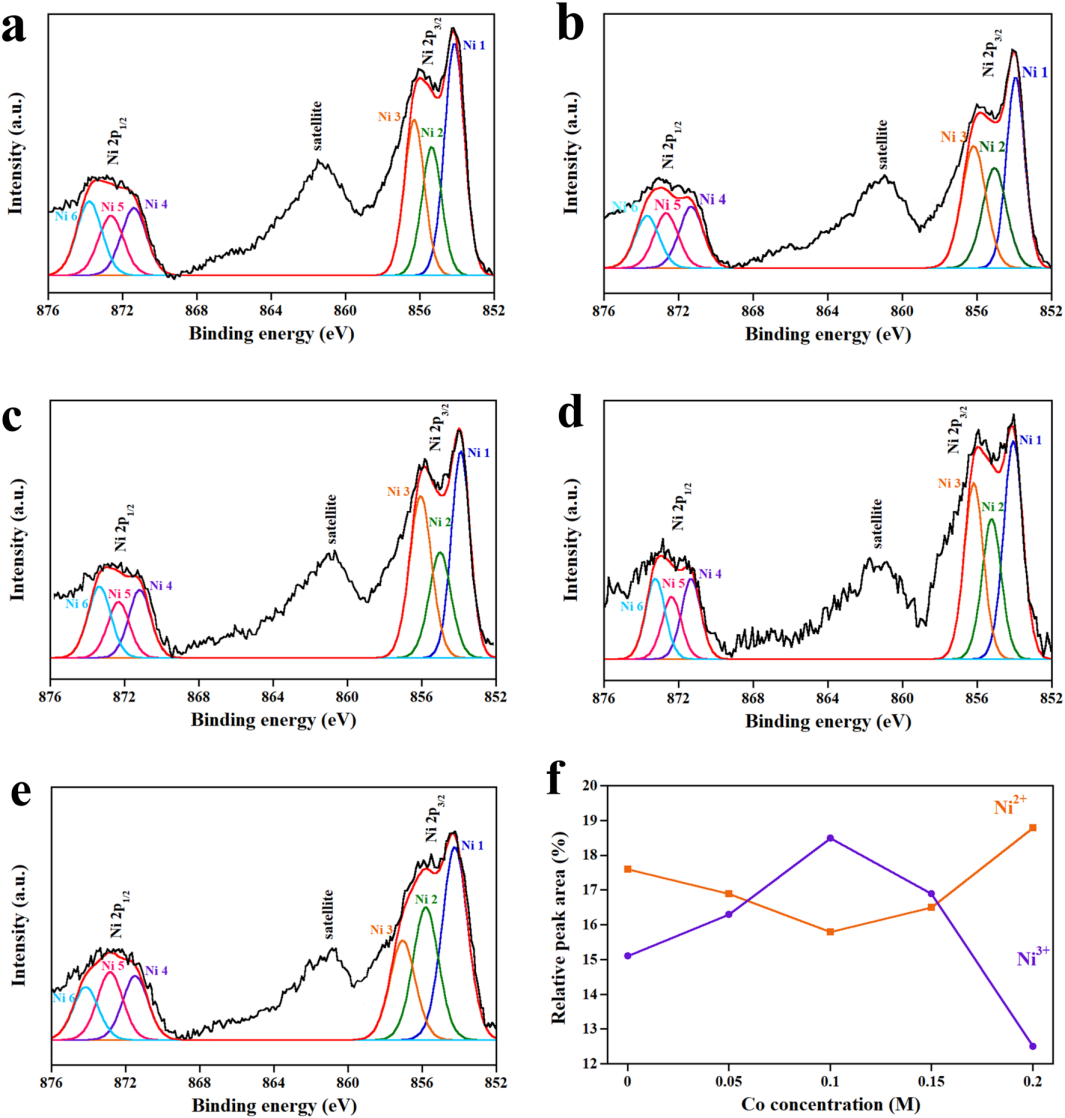
Ni 2p core level spectra of (**a**) 0 M, (**b**) 0.05 M, (**c**) 0.1 M, (**d**) 0.15 M and (**e**) 0.2 M Co-NiO film. (**f**) The relative peak area of Ni^2+^ and Ni^3+^ versus Co concentration.

**Figure 6:**
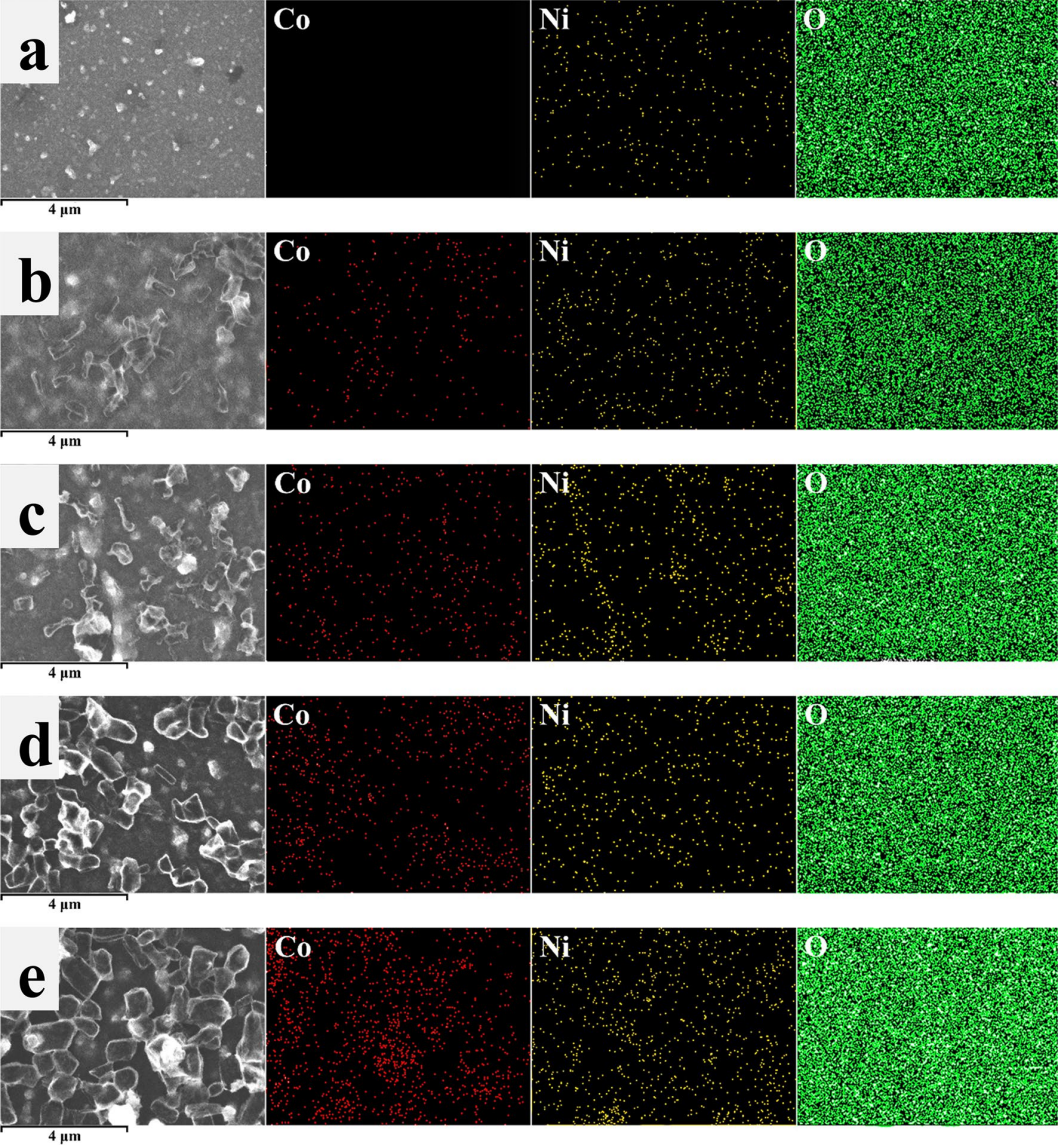
EDS mapping of (**a**) 0 M, (**b**) 0.05 M, (**c**) 0.1 M, (**d**) 0.15 M and (**e**) 0.2 M.

The chemical composition and the chemical bond of Co-NiO film were investigated by XPS. Note that the results of the investigation are displayed in Tables 1 and 2. Figure 4a illustrates the survey spectra of Co-NiO films, which reveals that the composition of the films is Ni, Co, O and C without other elementals. The appearance of carbon is caused by the contamination, which is commonly found in the air-exposed sample. As shown in Fig. 4b, the Co 2p core level spectrum contains the deconvoluted peaks of Co 1, Co 2, Co 3 and Co 4, which was assigned to Co^2+^ and Co^3+^^22–25^. The deconvolutions of the O 1s core level spectrum are shown in Fig. 4c. It was found that the peaks of O 1 and O 2 corresponded to O^2−^^26,27^. While the O 3 and O 4 peaks were in accordance with C(O)OH and O=C, respectively^28^. The C 1s core level spectrum displayed in Fig. 4d indicates five deconvoluted peaks of C 1, C 2, C 3, C 4 and C 5. These peaks were related to C–C, C–O, C=O, C=O and O=C–O, respectively^29–32^. To understand the influence of Co concentration on the quantity of Ni^3+^ and Ni^2+^, the Ni 2p core level spectrum of Co-NiO films was examined. As shown in Fig. 5a–e, it was found that the Ni 2p core level spectra of 0 M, 0.05 M, 0.1 M, 0.15 M and 0.2 M Co-NiO films consist of satellite and sublevel peaks of Ni 2p_3/2_ and Ni 2p_1/2_. These sublevel peaks were resolved into six peaks (Ni 1 to Ni 6), which Ni 1, Ni 2, Ni 4 and Ni 5 peaks were assigned to Ni^2+^ and the remaining peaks (Ni 3 and Ni 6) were registered to Ni^3+^^33,34^. The Ni 1 to Ni 6 peaks of 0 M, 0.05 M, 0.1 M, 0.15 M and 0.2 M Co-NiO films have a similar location but their intensity is different because the number of Ni^3+^ and Ni^2+^ ions in each condition is not equal, depending on Co concentration. With the increase in Co concentration, the Ni^3+^ ions in the NiO lattice increased as shown in Fig. 5f. The following decrease of the Ni^3+^ ions at the concentration of Co higher than 0.1 M was originated by the formation of CoO at the surface and grain boundaries of NiO, which act as carrier traps and prevent the substitution of Ni^2+^ ions by Co^2+^ ions. A similar explanation was found by other reports of doped-metal oxides^35,36^. Table 2 illustrates the chemical composition of Co-NiO films. It was found that the decrease in an atomic concentration (at%) of Ni 2p is affected by the increase in the content of Co, which is generally found in the report related to dopant^37^. To support the results of the chemical composition in Co-NiO films evaluated by XPS, the EDS was conducted. The results of the EDS demonstrate in Table 2. It has been observed that the values of Ni and Co measured by EDS are lower than the values from XPS. This is caused by receiving different information. For XPS, information on chemical composition comes from the surface of the film while EDS provides the information not only from the film but also from the glass substrate. Therefore, the quantity of Ni and Co elements are diminished by the chemical compositions of the glass substrate. Nevertheless, the percentage of change in at % of Ni and Co evaluated by EDS is similar to the result of XPS. Figure 6 presents the EDS mapping of Co-NiO films. For 0.05 M and 0.1 M, the Co, Ni and O elements are well distributed in the films while the distribution of Co at 0.15 M and 0.2 M is mostly found at the location of the submicron particles. This can be interpreted that the submicron particles are the CoO, which is in agreement with the result of XPS.

**Table 1:** XPS data of Co-NiO film.

Peak	Component	Position BE (eV)	FWHM (eV)	Assignment	References
Co 2p_3/2_	Co 1	780.2	2.5	Co^2+^	^22^
	Co 2	782.4	2.5	Co^2+^	^23^
Co 2p_1/2_	Co 3	795.3	2.5	Co^3+^	^24^
	Co 4	797.4	2.5	Co^2+^	^25^
O 1s	O 1	529.6	0.9	O^2-^	^26,27^
	O 2	531.1	1.4	O^2-^	^26,27^
	O 3	532.2	1.4	C(O)OH	^28^
	O 4	533.4	1.4	O=C	^28^
C 1s	C 1	284.9	1.1	C–C	^29^
	C 2	286.1	1.1	C–OH	^30^
	C 3	286.9	1.1	C=O	^31^
	C 4	288.3	1.2	C=O	^29^
	C 5	289.4	1.2	O=C–O	^32^
Ni 2p_3/2_	Ni 1	853.9	1.1	Ni^2+^	^33^
	Ni 2	855.1	1.5	Ni^2+^	^33^
	Ni 3	856.2	1.5	Ni^3+^	^33^
Ni 2p_1/2_	Ni 4	871.3	1.5	Ni^2+^	^34^
	Ni 5	872.7	1.5	Ni^2+^	^26^
	Ni 6	873.7	1.5	Ni^3+^	^34^

**Table 2:** The atomic concentration of the composition in Co-NiO films.

Composition (molarity, M)	XPS (at%)				EDS (at%)			
	Co 2p	Ni 2p	O 1s	Other	Co	Ni	O	Other
0.00	0.0	16.1	36.4	47.5	0.0	1.5	61.7	36.8
0.05	2.8	13.1	34.6	49.5	0.3	0.8	61.2	37.7
0.10	6.2	5.3	35.8	52.7	0.6	0.5	61.4	37.5
0.15	7.3	2.2	34.6	55.9	0.7	0.3	58.0	41.0
0.20	8.3	2.4	33.5	55.8	0.8	0.5	64.7	34.0

### Electrical properties of Co-NiO films

**Figure 7:**
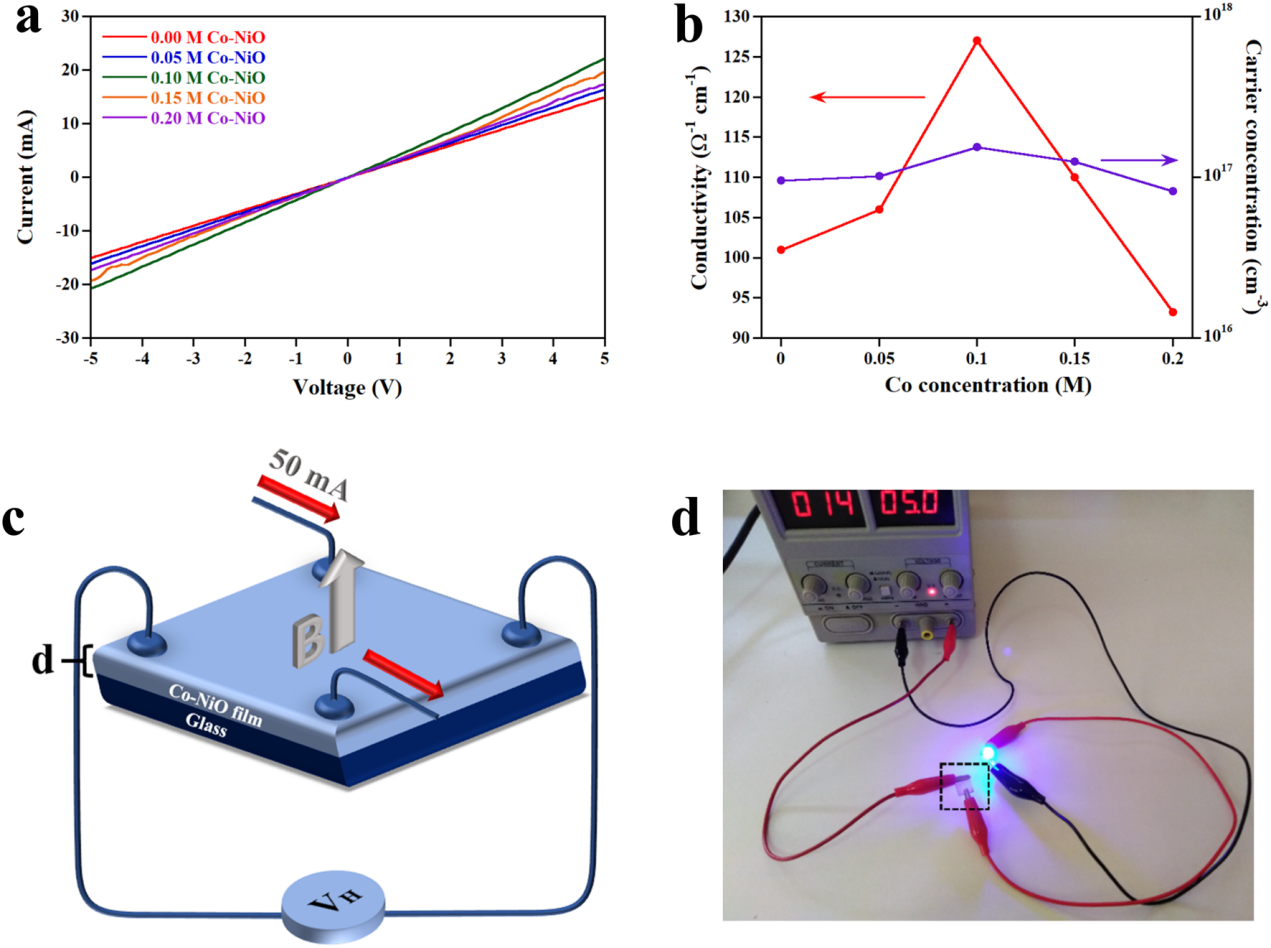
(**a**) I-V characteristic curves of samples. (**b**) The electrical conductivity and carrier concentration versus Co concentration. (**c**) Schematic diagram of van der Pauw method. (**d**) The practical use of 0.1 M Co-NiO film as a transparent conductor (in a black square).

The current–voltage (I-V) curves of samples are shown in Fig. 7a. It was observed that the I-V characteristics of samples are linear, reflecting an ohmic contract. The resistivity ($\rho$) of samples was calculated by the following equation^20^:

4 $$ \rho = R\frac{A}{L} $$

where *R* is the resistance, *A* is the area and *L* is the length between electrodes. As shown in Table 3, the decrease in resistivity from 9.9 × 10^–3^ to 7.89 × 10^–3^ Ω cm was caused by increasing the number of higher-valence Ni^3+^ due to the Co^2+^ substituted Ni^2+^^38^. According to this result, Co-NiO films fabricated by our method indicate the lower resistivity than Co-NiO films prepared by the solution methods^22,39^. It is well known that the resistivity is inversely related to the crystallite size^40^. Our method provides the film with two orders of magnitude bigger crystallite size than the films deposited by the solution method due to the influence of the uniform magnetic field on the sparking method as mentioned in our previous report^19,39^. At the higher concentration than 0.1 M, the resistivity of the films increased. This result is attributed to the increase of defects and the decrease of Ni^3+^, which is in agreement with the dislocation density and the carrier traps obtained by XRD and XPS. The electrical conductivity of Co-NiO film is shown in Fig. 7b. Obviously, the electrical conductivity of the films depends on the Co concentration, which is correlated with the resistivity in Table 3. To further investigate the electrical properties of Co-NiO film, Hall effect measured by the van der Pauw method was used. Note that the schematic diagram of the van der Pauw method is shown in Fig. 7c. The hall coefficient (*R*_*H*_) and the carrier concentration (*n*) of samples are given by the following equation^41^:

5 $$ R_{H} = \frac{{V_{H} d}}{IB} $$

6 $$ n = \frac{1}{{R_{H} e}} $$

**Table 3:** Concentration, band gap, resistivity, hall coefficient, carrier concentration and transmittance values of Co-NiO films.

Sample name	Band gap (eV)	Resistivity (Ω cm)	Hall coefficient (cm^3^/C)	Carrier concentration (cm^−3^)	Transmittance (400–700 nm) (%)
0 M Co-NiO	3.45	9.9 × 10^–3^	65.2	9.57 × 10^16^	93.32
0.05 M Co-NiO	3.33	9.43 × 10^–3^	61	1.02 × 10^17^	73.54
0.10 M Co-NiO	3.15	7.89 × 10^–3^	40.6	1.54 × 10^17^	58.73
0.15 M Co-NiO	3.06	9.1 × 10^–3^	50	1.25 × 10^17^	47.81
0.20 M Co-NiO	2.91	1.07 × 10^–2^	76	8.21 × 10^16^	41.34
Commercial ITO	–	4.34 × 10^–4^	− 0.54	1.16 × 10^19^	92.22

where *V*_*H*_ is the change in voltage, *d* is the thickness of film, *I* is the constant current (50 mA), *B* is the magnetic field (100 mT) and *e* is the elementary charge (1.602 × 10^–19^ C). The results of the calculation are demonstrated in Table 3. It was observed that the hall coefficient of all conditions of Co-NiO films is positive, revealing the characteristic of p-type semiconductors. Meanwhile, the increase in Co concentration from 0.05  to 0.1 M improves the carrier concentration of Co-NiO films. Nevertheless, the carrier concentration of Co-NiO films reduced at the concentration of Co higher than 0.1 M. This result corresponds to the increase of resistivity. To illustrate that the Co-NiO film by sparking under the uniform magnetic field can be practically used as the transparent conductor, 0.1 M Co-NiO film was connected with a blue light-emitting diode (LED) supplied by the voltage of 5 V and current of 14 mA as shown in Fig. 7d. The comparison between ITO and 0.1 M Co-NiO indicates that the resistivity and the carrier concentration of 0.1 M Co-NiO differ from ITO by ~ 1 to 2 orders of magnitude^42,43^.

## Conclusions

In this work, the Co-NiO film was successfully deposited by the two steps, which the first step is the deposition of NiO film by spark discharge under the uniform magnetic field and the second step is the spin coating of Co solution on NiO film. The increase in Co concentration from 0.05  to 0.1 M helps to improve the crystallinity and increases the carrier concentration of NiO film, resulting in a decrease of resistivity. This consequence is in agreement with the increase in the number of higher-valence Ni^3+^ because of the Co^2+^ substituted Ni^2+^, which confirms our assumption. By considering with optical and electrical properties, 0.05 M Co-NiO film is promising applications in TCO.

## Methods

### Preparation of Co solution

The 0.05 M, 0.1 M, 0.15 M and 0.2 M of cobalt acetate tetrahydrate (Co(CH_3_COO)_2_·4H_2_O, 99%, Ajax Finechem) were dissolved in 20 ml of absolute ethanol. The solution was stirred and refluxed at 80 °C for 10 min. After reflux, the solation was left at room temperature for 2 h, which leads to the formation of a pink solution^44^.

### Preparation of sparking under a uniform magnetic field

**Figure 8:**
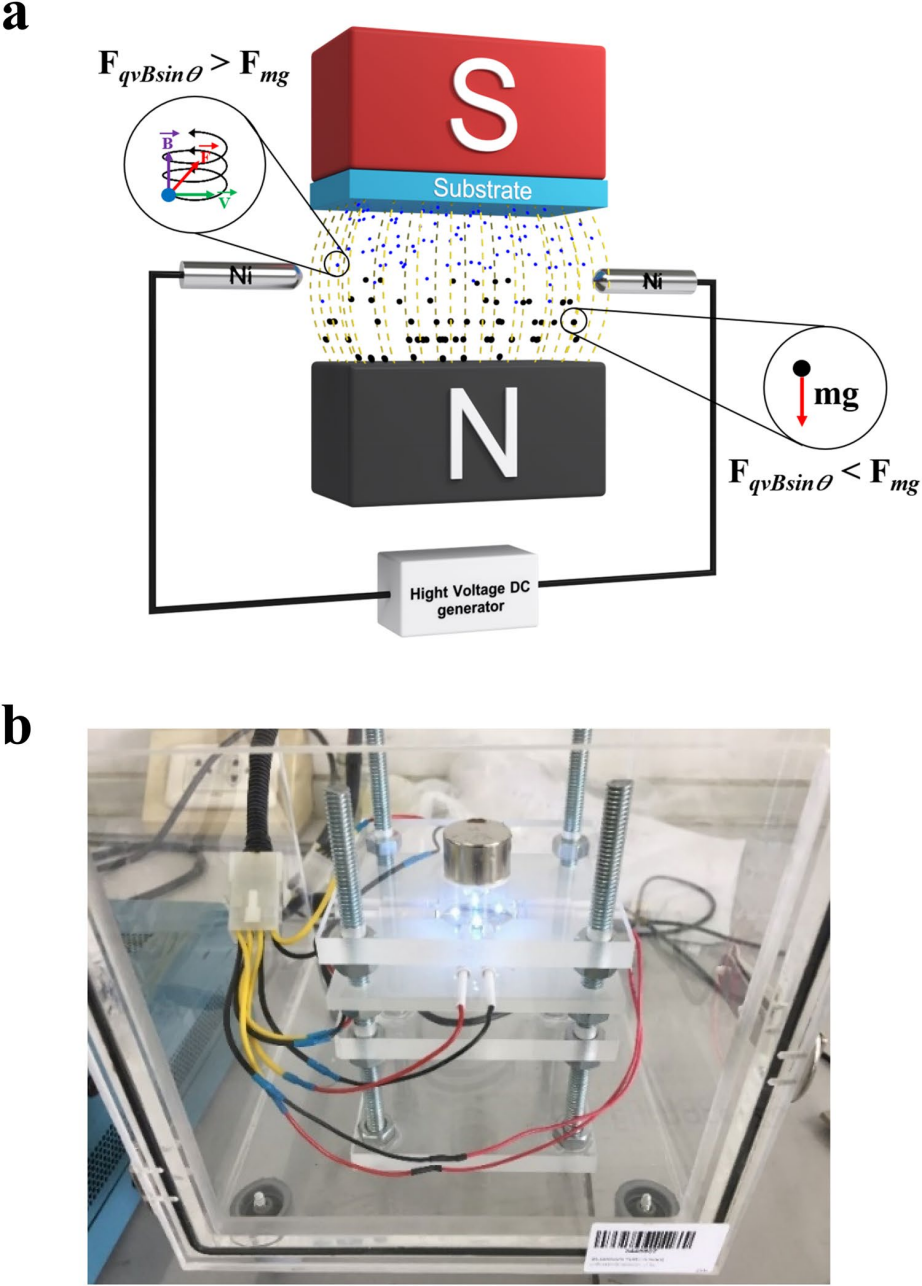
(**a**) Schematic diagram of the sparking method under a uniform magnetic field. (**b**) The practical use of spark discharge apparatus.

To separate the small particles from the big particles, the Lorentz force and the net force were used, which shows in Fig. 8. The spark discharge has four spark head. Each head consists of anode and cathode made of nickel wires (99.98%, Advent Research Materials Ltd) with a diameter of 0.5 mm. The distance between anode and cathode was 1 mm. The distance between the spark head and substrate was 5 mm. The operating voltage was approximately 3 kV. The pulse frequency of the sparking was 13 Hz. The spark discharge was operated under ambient air.

### Fabrication of Co-NiO film

**Figure 9:**
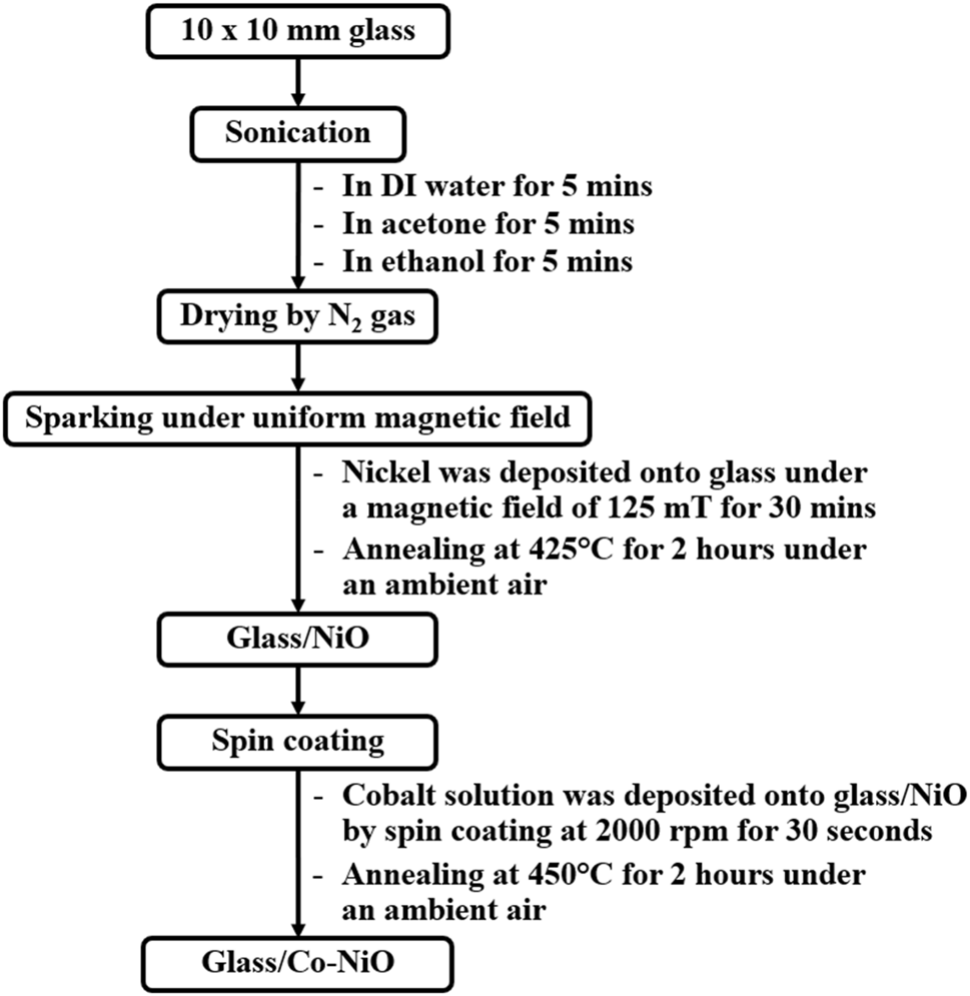
The flow chart for the fabrication of Co-NiO film.

The flow chart for the fabrication of Co-NiO film is shown in Fig. 9. The 10 × 10 mm^2^ glass samples were cleaned by sonication in distilled water, acetone and ethanol, respectively. After that, the glass samples were dried by flowing the N_2_ gas. The NiO was deposited onto the glass by sparking under a magnetic field of 125 mT for 30 min. Subsequently, the glass/NiO was annealed at 425 °C for 2 h on a hot plate magnetic stirrer. Note that the deposition and annealing were operated under ambient air. The Co solution was deposited onto glass/NiO by spin coating at 2000 rpm for 30 s. The samples were then annealed at 450 °C for 2 h under ambient air.

### Characterization

The samples were characterized by a scanning electron microscope (SEM, JSM-6335F), X-ray diffraction spectrometer (XRD, Philips X0Pert MPD), Atomic force microscope (AFM, Nanoscope IIIa, Veeco), UV–Vis spectrophotometer (Cary 50), ellipsometer (alpha-SE), X-ray photoelectron spectrometer (AXIS ULTRA^DLD^ XPS, Kratos analytical, UK), Energy dispersive x-ray spectrometer (EDS, Oxford Instrument) and source measure unit (Keithley 2450), respectively.
